# Wildfire and topography drive woody plant diversity in a Sky Island mountain range in the Southwest USA

**DOI:** 10.1002/ece3.8158

**Published:** 2021-10-05

**Authors:** Andrew M. Barton, Helen Poulos

**Affiliations:** ^1^ Department of Biology University of Maine at Farmington Farmington ME USA; ^2^ College of the Environment Wesleyan University Middletown CT USA

**Keywords:** alpha diversity, beta diversity, gamma diversity, intermediate disturbance hypothesis, Madrean, pyrodiversity, Sky Islands, species diversity, wildfire

## Abstract

**Aim:**

Drastic changes in fire regimes are altering plant communities, inspiring ecologists to better understand the relationship between fire and plant species diversity. We examined the impact of a 90,000‐ha wildfire on woody plant species diversity in an arid mountain range in southern Arizona, USA. We tested recent fire‐diversity hypotheses by addressing the impacts on diversity of fire severity, fire variability, historical fire regimes, and topography.

**Location:**

Chiricahua National Monument, Chiricahua Mountains, Arizona, USA, part of the Sky Islands of the US–Mexico borderlands.

**Taxon:**

Woody plant species.

**Methods:**

We sampled woody plant diversity in 138 plots before (2002–2003) and after (2017–2018) the 2011 Horseshoe Two Fire in three vegetation types and across fire severity and topographic gradients. We calculated gamma, alpha, and beta diversity and examined changes over time in burned versus unburned plots and the shapes of the relationships of diversity with fire severity and topography.

**Results:**

Alpha species richness declined, and beta and gamma diversity increased in burned but not unburned plots. Fire‐induced enhancement of gamma diversity was confined to low fire severity plots. Alpha diversity did not exhibit a clear continuous relationship with fire severity. Beta diversity was enhanced by variation in fire severity among plots and increased with fire severity up to very high severity, where it declined slightly.

**Main Conclusions:**

The results reject the intermediate disturbance hypothesis for alpha diversity but weakly support it for gamma diversity. Spatial variation in fire severity promoted variation among plant assemblages, supporting the pyrodiversity hypothesis. Long‐term drought probably amplified fire‐driven diversity changes. Despite the apparent benign impact of the fire on diversity, the replacement of two large conifer species with a suite of drought‐tolerant shrubs signals the potential loss of functional diversity, a pattern that may warrant restoration efforts to retain these important compositional elements.

## Graphical abstract

In an arid mountain range in southern Arizona, USA, alpha species richness declined, while beta and gamma diversity increased in burned but not unburned plots. The results reject the intermediate disturbance hypothesis for alpha diversity and support the role of pyrodiversity in beta diversity. Despite the apparent benign impact of the fire on diversity, the replacement of two large conifer species with shrubs signals the potential loss of functional diversity.

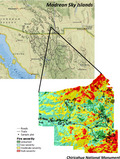

## 1 INTRODUCTION

Fire plays a key role in ecosystems across the Earth, influencing species composition, physical structure, and processes (Archibald et al., 2018; Bond et al., 2005; Bowman et al., 2020; Krawchuk et al., 2009; Pausas & Keeley, 2009). Natural disturbances such as fire have long been recognized as regulators of biological diversity (Connell, 1978; Huston, 1994). Understanding spatial and temporal variation in the fire‐diversity nexus is especially critical given the profound anthropogenic alterations of fire regimes across the Earth and their impacts on ecosystems and species, including humans (Bowman et al., 2020; Coop et al., 2020). Ecologists have responded by intensifying their efforts to develop generalizations about species diversity and fire that address the challenges of a fierier world in the Anthropocene (e.g., Bowman et al., 2020; Burkle et al., 2015; Coop et al., 2020; Enright et al., 2015; He et al., 2019; Miller & Safford, 2020; Pausas & Ribeiro, 2017; Perry et al., 2011).

Whittaker (1970, 1972) partitioned species diversity into three components: alpha diversity (α), beta diversity (β), and gamma diversity (γ). α is species diversity at a point in the landscape (i.e., a single “local” site), which itself can be decomposed into the number of species (richness) and the evenness of abundances among species. α is usually measured as the mean or median diversity of multiple sites found within an area of study. β captures differences in species assemblages among sites and has been measured with a wide variety of approaches (Anderson et al., 2011). The combination of α and β produces γ, the total species diversity supported in the larger area: landscape diversity. Although myriad hypotheses have been proposed and tested regarding the relationship between fire and these three levels of diversity, generalizations have been elusive, which signals the need for further conceptual work and hypothesis testing for understanding the variety of ways in which fire influences diversity (Anderson et al., 2011; Burkle et al., 2015; He et al., 2019; Kelly & Brotons, 2017; Miller & Safford, 2020; Parr & Andersen, 2006). In this paper, we test key hypotheses on the impact of wildfire on woody plant diversity in a topographically complex mountain range.

The intermediate disturbance hypothesis (IDH) proposes that species richness—usually α, less commonly β and γ—varies predictably with disturbance gradients in a unimodal, hump‐shaped fashion, in which intermediate levels of disturbance intensity or frequency maintain high diversity (Connell, 1978; Sousa, 1979). The IDH has been vigorously debated (Fox, 2013; Huston, 2014), with mixed support across a wide range of disturbance types and taxa (Sheil & Burslem, 2013). Nevertheless, research has repeatedly revealed a hump‐shaped relationship between plant species richness and fire severity (DeSiervo et al., 2015; He et al., 2019; Miller & Safford, 2020; Richter et al., 2019; Strand et al., 2019), especially in frequent, low‐severity fire regimes (Miller & Safford, 2020). The assumed underlying mechanisms vary, but most propose that different fire severities environmentally select for different sets of species. Under no fire or low fire severity, for example, competitive and fire‐resistant species should thrive, whereas, after a high‐severity fire, fast‐growing, rapidly colonizing species should predominate. The IDH proposes that at intermediate fire severity, both sets of species can coexist, resulting in a peak in species richness. Despite support for the IDH for fire, other studies have detected neutral, linear positive, and negative relationships between species diversity and fire severity (He et al., 2019; Miller & Safford, 2020).

Martin and Sapsis (1992) coined the term “pyrodiversity” to capture a growing awareness of the ecological importance of variation across landscapes in fire severity, frequency, size, and other attributes (see also Bowman et al., 2016; He et al., 2019; Krawchuk & Moritz, 2011; Perry et al., 2011). They argued that pyrodiversity promotes variation in plant assemblages among sites (i.e., β) because, as with the underlying assumption of the IDH, different sets of species thrive under different conditions related to fire, a phenomenon observed for decades in fire‐prone ecosystems (Bond et al., 2005; Pausas & Ribeiro, 2017; Romme, 1982). Mixed‐severity fire regimes, for example, provide a complex mosaic of postfire conditions that should support a wider range of plant species across a landscape than would a narrower low‐ or high‐severity fire regime alone. These arguments promoted an emerging management dictum that prescribed burning aimed at fostering biodiversity (β and γ) should create a broad spectrum of fire patch characteristics to provide conditions required for the regeneration and persistence of a diverse range of native biota (Bowman et al., 2016; Kelly & Brotons, 2017; Perry et al., 2011). Adding patches of fire to an otherwise long unburned but fire‐prone area will generally enhance the diversity of most taxa (He et al., 2019), but there is disagreement about the rigor of field studies, the shape of the relationship between fire and biodiversity, support for underlying assumptions, and the strength of the evidence for broadly applying these ideas to land management (Bowman et al., 2016; Kelly & Brotons, 2017; Parr & Andersen, 2006; Perry et al., 2011).

Miller and Safford (2020) argue that the IDH and the pyrodiversity hypotheses largely ignore the interaction between life history traits and the historical fire regime of particular ecosystems. They propose that the historical fire regime acts as a filter, selecting only those species with the capacity to regenerate and persist under those conditions. Such life history traits shaped over evolutionary time to adapt to the prevailing fire regime are unlikely to confer similar success to fire regimes other than the historical one. As an example, fire‐resistant tree species, with thick insulative bark and regeneration from seed, perform well and often dominate under frequent, surface fire regimes, but are readily killed in ecosystems with infrequent, stand‐replacing fires (Barton & Poulos, 2018; Coop et al., 2020). This leads to the hypotheses that α richness and β should peak at the historical fire severity of an ecosystem rather than necessarily at intermediate severity as predicted by the IDH. It predicts further that adding patches of fire outside of the historical fire regime will not necessarily promote variation in species assemblages across sites (i.e., β) or total landscape diversity (γ) (Miller & Safford, 2020).

In an effort to further explore the relationships between landscape variation in fire severity and woody plant species diversity, we examined the impact of a large 2011 wildfire on α, β, and γ in an arid, fire‐prone mosaic of shrub, woodland, and forest ecosystems in Chiricahua National Monument in the Sky Islands of Arizona, USA. Before Euro‐American settlement (<1,890), frequent surface fires predominated in pine and pine–oak forests (Barton et al., 2001; Kaib et al., 1996; Swetnam & Baisan, 1996; Swetnam et al., 1989, 2001), whereas more arid woodlands and interior chaparral experienced mixed fire regimes with longer fire intervals (Baisan & Morino, 2000; Kaib et al., 1996). Starting in the late 1800s, reduction in fine fuel by livestock grazing and then active fire suppression greatly reduced wildfire incidence for more than a century (Leopold, 1924; Marshall, 1957; Swetnam et al., 2001). This led to substantial increases in tree densities and woody plant cover, including fire‐sensitive species (Baisan & Morino, 2000; Taylor et al., 2021). Higher fuel loads combined with an increasingly warmer and drier climate led to the 2011 Horseshoe Two Fire, which burned ~90,000 ha across the entire mountain range, a size unprecedented in the historical fire record, part of a surge in very large fires with a significant high‐severity component in the western USA (Abatzoglou & Williams, 2016; Dennison et al., 2014; Dillon et al., 2011; Singleton et al., 2019; Westerling, 2016).

The Horseshoe Two Fire offered the opportunity to evaluate temporal shifts in woody plant diversity across a range of fire severities, spanning from unburned to high‐severity wildfire. To this end, we sampled woody plant diversity in 138 plots before (2002–2003) and after (2017–2018) the 2011 fire in three vegetation types and spanning wide fire severity and topographic gradients. We specifically addressed (1) whether α, β, and γ changed from the pre‐ to postfire sample periods, (2) the extent to which these changes were driven by the Horseshoe Two Fire, (3) the direction and shape of the relationship of α, β, and γ to fire severity and fire variability among plots, (4) whether diversity patterns with respect to fire were tied to the underlying historical fire regimes of the three vegetation types, and (5) the role of topography in shaping biodiversity independent of the Horseshoe Two Fire.

## 2 METHODS

### 2.1 Study area

**FIGURE 1:**
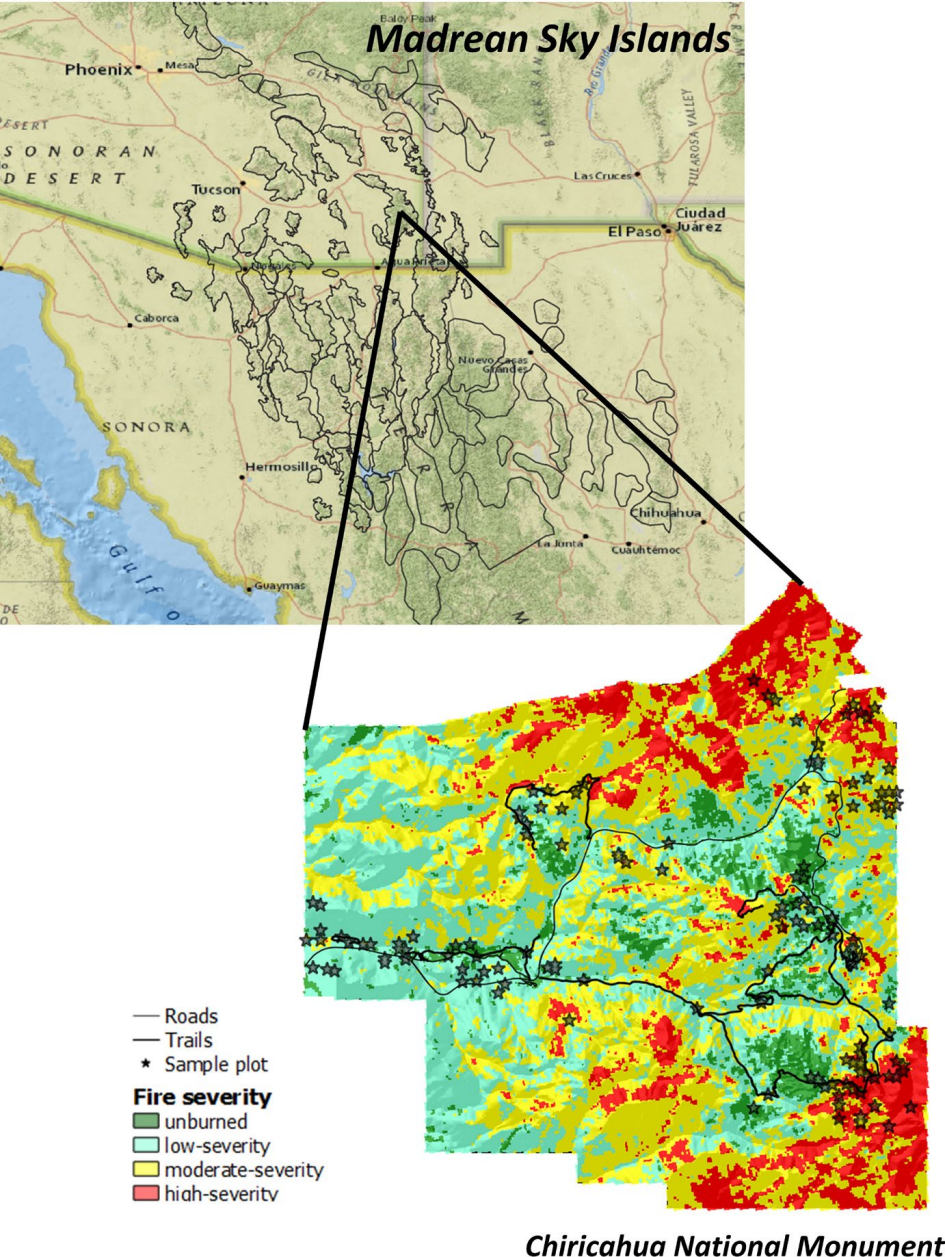
Map of Chiricahua National Monument, Cochise County, Arizona, USA, set into the regional geography of the Sky Islands in the US–Mexico borderlands. Vegetation plot locations are indicated with stars, and fire severity is depicted with different colors

Located in southeastern Arizona (32°00′20” N, 109°21′24” W), Chiricahua National Monument (CHIR) encompasses 4,850 ha in the Chiricahua Mountains (Figure 1), which are part of the Sky Islands, an archipelago‐like northern extension of the Mexican Sierra Madre Occidental (DeBano, 1995). Elevations in CHIR range from 1,562 to 2,228 m a. s. l. Soils are shallow and derived mainly from volcanic rhyolites and monzonites deposited in the early‐ to mid‐Miocene, although pre‐Tertiary rock is prominent at lower elevations (Drewes, 1973). The terrain of CHIR varies from level desert grassland to highly dissected, rocky uplands with steep‐walled canyons and incised towers.

The climate is semiarid (annual precipitation $\bar{X}$ = 490.2 mm), typically with a dry season from April to June ($\bar{X}$ = 42.7 mm) and a rainy season from July to September ($\bar{X}$ = 251.5 mm), driven by the North American Monsoon System (Adams & Comrie, 1997). Near the CHIR visitor center at 1,650 m a.s.l., mean minimum and maximum temperatures are $\bar{X}$ = −1.2°C and 13.4°C, respectively, for January and $\bar{X}$ = 15.5 and 31.8°C, respectively, for July. From low to high elevation, temperature decreases and moisture availability increases because of the combined effects of reduced evaporative demand and orographic lifting of moisture‐laden air (Barton, 1994; Shreve, 1915; Vivoni et al., 2007; Whittaker et al., 1968; Whittaker & Niering, 1975).

The Sky Islands of the US–Mexico borderlands support high levels of biodiversity and endemism, a product of the mixing of continental biomes and an isolated location midway between tropical and temperate regions (DeBano, 1995; Gehlbach, 1981; Poulos et al., 2007; Whittaker & Niering, 1975). A rich diversity of plant communities, classified in a variety of ways, occur in CHIR, including semi‐desert grassland, interior chaparral, piñon–oak–juniper woodlands, pine–oak woodland, pine forest, and mixed conifer forest (Barton, 1994; Bennett et al., 1996; Poulos et al., 2007; Roseberry & Dole, 1939; Reeves, 1976; Taylor et al., 2021). Plant nomenclature in this study followed the USDA Plants Database (USDA & NRCS, 2021), with aid from Bennett et al. (1996). Given the widespread occurrence of nonwoody plants in the study area, the results presented here represent only a portion of the entire set of plant species and communities in CHIR.

Historical fire regimes (<1,890) on the land that is now Chiricahua National Monument differed across the three main vegetation types investigated here (see Section 2.2 for a depiction of those). Madrean pine–oak forests, which occur primarily in canyons and at intermediate elevations, supported a frequent, low‐severity, surface fire regime, with a mean fire return interval of about 2 to 15 years (Baisan & Morino, 2000; Barton et al., 2001; Kaib et al., 1996; Swetnam & Baisan, 1996; Swetnam et al., 1989, 2001). In contrast, piñon woodland and juniper woodland, which occur primarily on drier, lower elevation sites, were characterized by mixed‐severity fire regimes, including infrequent, stand‐replacing fires (Baisan & Morino, 2000; Taylor et al., 2021; Villarreal et al., 2020).

As a result of intensive livestock grazing and then active fire suppression (Leopold, 1924; Swetnam et al., 2001), the incidence of wildfires declined drastically in the Chiricahua Mountains after the 1880s, leading to a pervasive accumulation of live and dead fuels (Baisan & Morino, 2000; Taylor et al., 2021). Similarly altered fire regimes have been well documented for many of the Sky Islands in Arizona (O’Connor et al., 2014; Swetnam et al., 2001), whereas anthropogenic shifts in fire regimes have been less pronounced on the Mexico side of the US–Mexico border (Meunier et al., 2014; Villarreal et al., 2019, 2020).

During the extremely dry year of 2011 (Williams et al., 2014), the Horseshoe Two Fire burned ~90,000 ha, nearly 3/4 of the mountain range (Arechederra‐Romero, 2012). Within the boundaries of CHIR, 7% of the land was unburned, whereas 36%, 41%, and 16% burned at low, moderate, and high severity, respectively. The percentage for high‐severity fire is far higher and that for low severity far lower than the historical range of variation for Madrean pine–oak forests (Swetnam & Baisan, 1996; Swetnam et al., 2001; see Villarreal et al., 2020, for estimates). For piñon and juniper woodlands, however, the pattern of the Horseshoe Two Fire may be within the historical range, with precedents (e.g., 1886) for large wildfires, likely with a substantial high‐severity component (Baisan & Morino, 2000; see also Baker & Shinneman, 2004 and Villarreal et al., 2020).

### 2.2 Field methods and calculation of independent variables

To examine changes in species diversity from before to after the 2011 Horseshoe Two Fire, we remeasured vegetation during the summers of 2017 and 2018 in 138 plots established and first measured in 2002 and 2003 (Poulos et al., 2007; Figure 1). The distribution of plots was initially stratified using vegetation cover types (Kluber, 2000; Poulos et al., 2007; Taylor et al., 2021). Sample points were placed in the center point of homogeneous areas of a cover type larger than 1,800 m^2^. Highly dissected, rough terrain with vertical rhyolitic towers made random or systematic sampling impossible, as we were limited to sites <30° slope that were accessible by foot. Nevertheless, the plot network spanned gradients of vegetation types, topography, and fire severity arising from the Horseshoe Two Fire. None of the plots used in this study occurred within the perimeters of past US Park Service prescribed fires or the rare wildfire occurring within the park.

In the initial 2002–2003 survey, woody vegetation at each selected point was sampled in 5 × 25 m belt transects established parallel to the slope contour. The location (GPS) and azimuth (º) of each belt transect were recorded. We measured the basal diameter of all shrubs and trees of each genet ≥10 cm, counted individual juvenile plants (<10 cm basal diameter), tallied shrubs including cacti (stems), and estimated percent cover of each woody species in one of six cover classes (<1, 1–4, 5–24, 25–49, 50–74, and 75%–100%). Postfire plot remeasurements in 2017–2018 were identical to those employed in 2002–2003 in the same 5 x 25 m belt transects.

Using Ward clustering with NbClust (3.0) in the vegan package (2.5–7) in R (R Core Team, 2020), 138 plots were clustered into three prefire vegetation types: juniper woodlands (*n* = 59), piñon woodlands (*n* = 39), and pine–oak forest (*n* = 40). Juniper woodlands were characterized by alligator juniper (*Juniperus deppeana* Steud.), Emory oak (*Quercus emoryi* Torr.), three‐leaved sumac (*Rhus trilobata* Nutt.), Palmer's century plant (*Agave palmeri* Engelm.), catclaw mimosa (*Mimosa aculeaticarpa* Benth.), and twistspine pricklypear (*Opuntia macrorhiza* Englem.). Piñon woodlands were dominated primarily by border piñon (*Pinus discolor* Bailey and Hawksw), Toumey oak (*Q. toumeyi* Sarg.), pointleaf manzanita (*Arctostaphylos pungens* Kunth), Wheeler's sotol (*Dasylirion wheeleri* Wats.), Garry's silktassel (*Garrya wrightii* Torr.), and sacahuista (*Nolina macrocarpa* S. Watson). Pine–oak forests were composed mainly of Chihuahua pine (*P. leiophylla* var. *chihuahuana* (Engelm.) Shaw), Apache pine (*P. engelmannii* Carr.), Arizona pine (*P. arizonica* Engelm.), Arizona white oak (*Q. arizonica* Sarg.), silverleaf oak (*Q. hypoleucoides* Camus), Arizona madrone (*Arbutus arizonica* (Gray) Sarg.), and Douglas‐fir (*Pseudotsuga menziesii* (Mirb.) Franco).

We estimated fire severity of the 2011 Horseshoe Two Fire for each of the 138 plots using raster delta normalized burn ratio (dNBR; Eidenshink et al., 2007), a Landsat ETM+‐derived product that estimates change in fire severity from before to after a fire. The normalized burn ratio is calculated from ETM + bands 4 and 7 as (ETM4–ETM7)/(ETM4 + ETM7); ETM4 represents the near‐infrared spectral range (0.76–0.90 μm), and ETM7, the shortwave infrared spectral range (2.08–2.35 μm). Differenced NBR images (prefire NBR minus postfire NBR) are referred to as dNBR images. Prefire Landsat ETM + images are from the month before the fire, and postfire images are from 6 months after the fire for dNBR calculation. We acquired dNBR data from the Monitoring Trends in Burn Severity data distribution site (https://www.mtbs.gov/), extracting a value for each plot with the point sampling tool in QGIS (QGIS Development Team, 2020). In some cases, dNBR was used as a continuous independent variable; in others, dNBR fire severity classes (none, low, moderate, and high; MTBS, www.mtbs.gov) were employed for analyses.

We used elevation and the topographic relative moisture index (TRMI; Parker, 1982) as independent woody plant diversity predictors, as past research has revealed their importance in regulating woody plant species composition (Poulos et al., 2007). Elevation was extracted in QGIS (QGIS Development Team, 2020) for each plot from 30‐m resolution digital elevation models (DEMs) (https://lpdaac.usgs.gov); TRMI was calculated from field‐measured topographic position (ridge, upper elevation, midelevation, lower elevation, and valley), slope direction (in degrees), slope steepness (in degrees), and surface shape (convex, convex‐straight, straight, concave‐straight, and concave). TRMI provides a quantitative xeric to mesic continuum among plots, independent of elevation. We also extracted and used the terrain ruggedness index (TRI) from the 30‐m DEMS in QGIS, which is defined as the mean difference between a central plot pixel and its surrounding 8 pixels.

### 2.3 Statistical analysis

#### 2.3.1 General approach

We analyzed γ, α, and β for (1) changes over the two sample periods for all plots, for each vegetation type separately, and for burned versus unburned plots separately and (2) their continuous relationships with dNBR and the three topographic variables. Where interaction terms were integral to an explicit hypothesis, we retained significant and insignificant interactions; otherwise, insignificant interactions were dropped from models. For continuous independent variables, we first tested second‐degree polynomial models, dropping the quadratic term when it was insignificant; first‐order terms were retained in cases of a significant quadratic component. Separate from the diversity analyses, we used correlation analysis to identify relationships among gradients of fire severity (dNBR), elevation, TRMI, and TRI.

#### 2.3.2 Gamma diversity

For landscape diversity (γ), we used rarefaction (“EstimateS” 9.10; Colwell & Elsensohn, 2014) to estimate species richness (with 95% confidence intervals) and test for changes in γ from prefire to postfire for all plots combined and for each of the three vegetation types separately. Rarefaction addresses incomplete sampling of biota by extrapolating species richness out to a presumed asymptote where additional sampling would not discover any new species. We tested whether temporal change in γ was tied to the Horseshoe Two Fire and, if so, at which fire severities, by analyzing each of the four fire severity classes (no fire and low, moderate, and high severity). If the fire drove temporal changes, we would expect change for one or more of the classes of burned plots but not for unburned plots.

#### 2.3.3 Alpha diversity

For α, we used the “vegan” package (Oksanen et al., 2015) in R (R Core Team, 2020) to calculate species richness, evenness, inverse Simpson diversity index, Shannon–Weiner diversity index, Simpson index, and unbiased Simpson index for each plot for before and after the Horseshoe Two Fire. We used the paired *t* tests (in R) to compare 2002–2003 versus 2017–2018 α metrics for all plots combined and for each of the three vegetation types separately.

We tested whether temporal changes in α could be attributed to fire and, if so, at which severities, with paired *t*‐tests for sets of plots that did not burn and those that experienced low‐, moderate‐, and high‐severity fire. We used “lme4” (Bates et al., 2012) and “lmertest” (Kuznetsova et al., 2020) in R to test linear and polynomial mixed‐effects models for the influence of fire severity as a continuous variable (dNBR) on α and the interaction between timestep and dNBR, with the prediction that fire severity would affect α under postfire conditions only. Mixed‐effects models were used to account for nesting across time within plots. We examined whether vegetation types differed in the shape (linear, hump‐shaped, etc.) of the species richness–fire severity relationship by running linear and polynomial regressions of the change in the number of species over time for each plot versus dNBR for each vegetation type separately.

We used linear and polynomial mixed‐effects models to examine the relationship of α metrics with the topographic variables, elevation, TRMI, and TRI. The interaction term (timestep*topographic variable) of these models tested for shifts in these relationships from the prefire to postfire period.

#### 2.3.4 Beta diversity

For β, we used “vegan,” “betapart” (Baselga & Orme, 2012), and “ecodist” (Goslee & Urban, 2007, 2020) in R to calculate a variety of Sorensen dissimilarity statistics among plots on species presence–absence matrices. We partitioned total β into nestedness and turnover components (Baselga, 2010, 2012) to assess which process best explained spatiotemporal patterns. Nestedness occurs when communities with smaller numbers of species are subsets of richer ones, whereas species turnover refers to the replacement of some species with others (Baselga, 2010, 2012). Where appropriate, we tested whether a second‐degree polynomial model provided a better fit than a linear one for continuous independent variables.

We used three different approaches to test hypotheses about β—all focused on the roles of time, fire, vegetation types, and topography. First, we calculated pairwise dissimilarities among all plots (“pairwise plots”; mission V4 of Anderson 2011) for the prefire and the postfire sample periods separately. We tested hypotheses with these dependent variables using the adonis2 test in vegan, which employs the permutational MANOVA approach of McArdle and Anderson (2001). Classical statistical tests were inappropriate for these data because of the lack of independence among pairwise plot dissimilarities. We statistically tested for changes in total β, species turnover, and nestedness across the two sample periods for all plots and for each of the three vegetation types separately. To assess the extent to which the Horseshoe Two Fire drove these temporal changes in β, we separately analyzed plots that did not burn (control) versus those that experienced low‐, moderate‐, and high‐severity fire. Finally, we used the adonis2 test to develop the best model for the role of elevation, TRMI, and TRI in controlling β.

As a second and complementary approach (Legendre & De Cáceres, 2013), we calculated prefire versus postfire dissimilarities separately for each plot (“matched plots”; mission T2 of Anderson et al., 2011). We used general linear mixed‐effects models (Hothorn et al., 2017) to test for differences in prefire–postfire dissimilarities among the three vegetation types, among the four fire severity categories, across dNBR, and with respect to topography (elevation, TRMI, and TRI). We examined whether vegetation types differed in the shape (linear, hump‐shaped, etc.) of the β‐fire severity relationship by running linear and polynomial regressions of dissimilarities for each plot versus dNBR for each vegetation type separately.

Finally, we used the Mantel tests to examine whether postfire plot dissimilarities in species presence were correlated with Euclidian distances for fire severity (dNBR) and each of the topographic variables (“Mantel tests”; mission T3 of Anderson et al., 2011). Additionally, we carried out a Mantel test on the relationship between a matrix of prefire minus postfire pairwise plot species dissimilarities versus the Euclidian dNBR distance matrix in order to assess whether temporal changes in plot species dissimilarities were positively related to variability among plots in fire severity. These Mantel tests of fire severity test the hypothesis that pyrodiversity promotes β. For these Mantel tests, we also evaluated whether any of the significant relationships could be explained simply by differences between plots in geographic distance. We calculated pairwise plot geographic distances using the Geographic Distance Matrix Generator (Ersts, 2020), which were then subjected to a Mantel test between beta diversity and distance and to partial Mantel tests using each independent variable with distance as a second explanatory variable.

We examined the overall contributions of α and β to γ for before versus after the fire using γ for all plots, mean α richness per plot, and β calculated as Whittaker's β (1972), β*_w_* = γ/α.

## 3 RESULTS

### 3.1 Relationships among independent variables

Fire severity (dNBR) increased with elevation (*r* = 0.43; *t* = 5.6, *p* < .001), increased with terrain ruggedness (TRI: *r* = 0.24; *t* = −3.8, *p* < .001), and decreased with the topographic relative moisture index, from relatively xeric to mesic plots (TRMI: *r* = −0.31; *t* = −2.9, *p* = .004). TRI increased with elevation (*r* = 0.23; *t* = 2.7, *p* = .008) and decreased with TRMI (*r* = −0.37; *t* = −4.6, *p* < .001). TRMI decreased with elevation (i.e., became more xeric; *r* = −0.52; *t* = −7.1, *p* < .001). Scatterplots of these relationships are provided in Figure S1. Fire severity was also significantly lower in valleys than at other topographic positions, with no significant differences among ridgetops and lower, middle, and upper slopes (results not shown).

### 3.2 Gamma diversity (γ)

**FIGURE 2:**
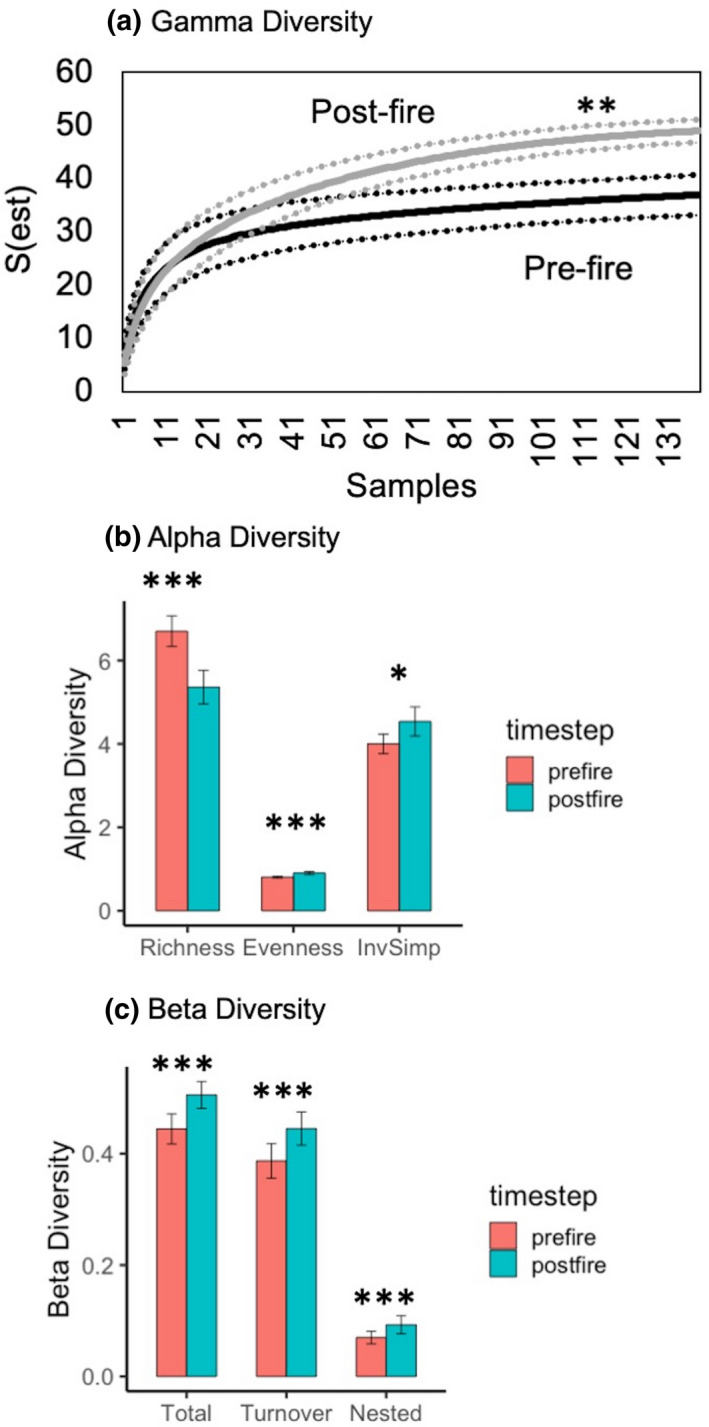
Differences for woody plant species between prefire and postfire sample periods in Chiricahua National Monument for (a) gamma diversity, showing rarefaction accumulation curve with estimate and 95% confidence internals, (b) mean (with 95% CI) for alpha diversity (species richness, species evenness, and inverse Simpson index, and (c) mean (with 95% CI) for beta diversity (total, species turnover, and nestedness, using Sorensen dissimilarity). Statistical significance: *p < .05, **p < .01, and ***p < .001

**FIGURE 3:**
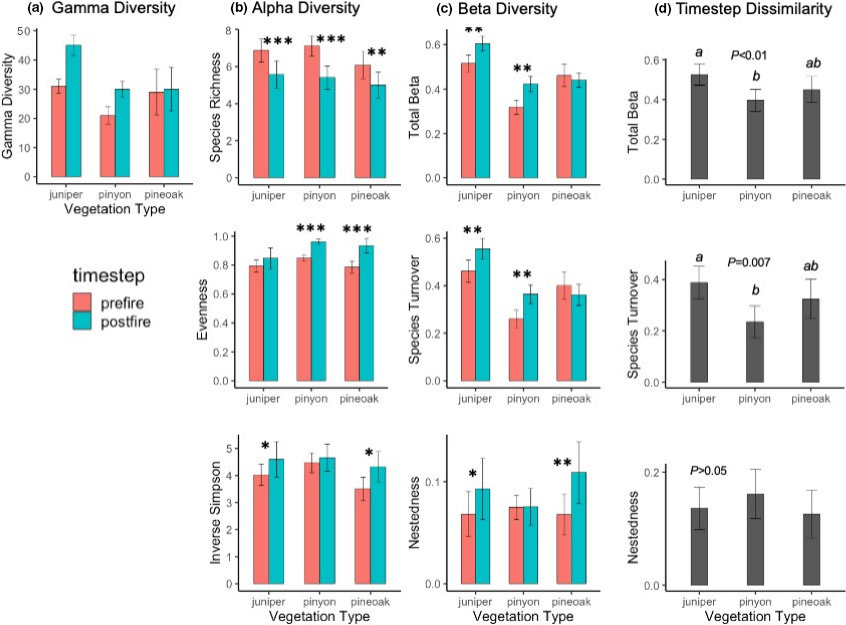
Changes for each of three vegetation types (total of 138 plots) in Chiricahua National Monument from the prefire to the postfire sample periods for (a) gamma diversity (from rarefaction with 95% CI), (b) mean (with 95% CI) alpha diversity metrics (species richness, species evenness, and the inverse Simpson index), (c) mean (with 95% CI) beta diversity expressed as all plot pairwise dissimilarities (Sorensen) for prefire and postfire species presence, and (d) beta diversity expressed as prefire/postfire dissimilarity for each plot independent of other plots. Asterisks (*p < .05, **p < .01, and ***p < .001) show prefire/postfire differences from paired *t* tests. For (d), *p*‐value is for different among vegetation types, and bars with different letters are significantly different from each other (*p* < .05)

**FIGURE 4:**
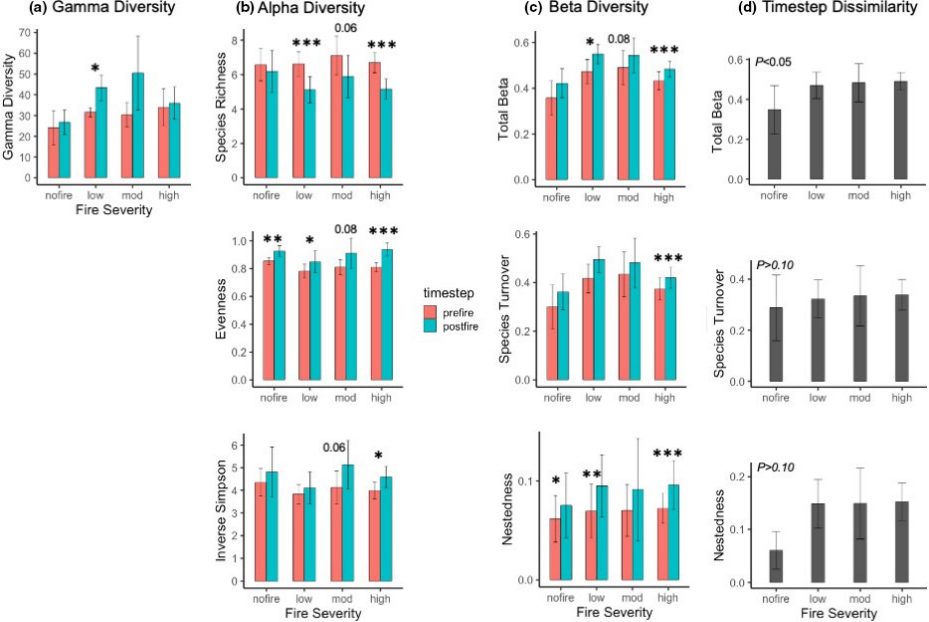
Changes for woody plant species in Chiricahua National Monument from the prefire to the postfire sample periods for 138 plots separately for each level of fire severity for (a) gamma diversity (from rarefaction with 95% CI), (b) mean (with 95% CI) for alpha diversity metrics (species richness, species evenness, and the inverse Simpson index), (c) mean (with 95% CI) for beta diversity expressed as all plot pairwise dissimilarities (Sorensen) for prefire and postfire species presence, and (d) beta diversity expressed as prefire/postfire dissimilarity for each plot independent of other plots. Asterisks (*p < .05, **p < .01, and ***p < .001) show prefire/postfire differences from paired *t* tests. For (d) only, *p*‐value within each bar is for all plots that burned versus those that did not burn. Exact *p*‐values are given for tests producing marginally insignificant results

We recorded 36 species in the 138 plots in 2002–2003 and 48 in 2017–2018, 6–7 years after the 2011 Horseshoe Two Fire. All species were native plants. The rarefaction estimate was 37 and 49 species, respectively, indicating a significant temporal increase in landscape woody plant richness (*p* < .01; Figure 2). Rarefaction‐calculated γ richness increased over time for juniper and piñon woodlands but not for pine–oak forest (Figure 3). If the temporal change in γ richness was tied to fire, then we would expect the increase to occur in burned plots only—a prediction supported by the results, with γ increasing significantly at low fire severity and marginally insignificantly at moderate fire severity (Figure 4).

Only three species were unique to the prefire data, whereas 13 were unique to the postfire sample. Species new to the 138 plots during the second sampling were cacti and shrub growth forms (e.g., *Amorpha fruticose* L., *Bouvardia ternifolia* (Cav.) Schltdl, *Philadelphus microphyllus* A. Gray, and *Toxicodendron rydbergii* (Small ex Rydb.) Green)), species typically found in open areas. In contrast, two of the three species disappearing from the plots during this time span—*Pinus arizonica* and *Pseudotsuga menziesii*—were coniferous tree growth forms that regenerate from seed. These were present elsewhere in the study area during the postfire sample, but no longer in the sample plots.

### 3.3 Alpha diversity (α)

The mean number of species per plot was 6.70 (±0.02 1*SE*) before and 5.36 (±0.03 1*SE*) after the fire. The Shannon–Weiner, Simpson, and unbiased Simpson indices did not change from pre‐ to postfire, and temporal change for these metrics was not associated with vegetation type, fire severity, or topographic position (*p* > .05; results not shown). Figures 2, 3, 4, Table 1, and Figures S2 and S3 display the results for species richness, evenness, and the inverse Simpson index, all of which exhibited significant patterns across space and time.

**TABLE 1:** Statistical results for linear mixed‐effects models of alpha diversity metrics (species richness, evenness, and inverse Simpson) in relation to fire severity (dNBR) and the topographic variables elevation, topographic relative moisture index (TRMI), and terrain ruggedness index (TRI). Analyzed data were abundance for each species in each of 138 plots. Results are provided for final models, factors, *t*‐values, and statistical significance (*p < .05, **p < .01, and ***p < .001)

Model	Factor	Species richness	Evenness	Inverse Simpson
Timestep +fire severity	dNBR	3.7***	2.9***	4.4**
	dNBR^2^	−4.7***	−2.2*	−4.8***
	Timestep	2.0*	−0.6	−1.1
	Timestep*dNBR	1.6	−2.8**	−0.5
Timestep +topography	Elevation	5.3***	3.4***	4.8***
	Elevation^2^	−5.4***	−3.4***	−4.9***
	TRMI	−0.7	1.3	1.5
	TRI	3.2***	2.2*	3.6***
	TRI^2^	−2.2*	−2.2*	−2.2*
	Timestep*Elevation	NS	NS	NS
	Timestep*TRMI	NS	NS	NS
	Timestep*TRI	NS	NS	3.0***

Mean species richness per plot decreased significantly from the first to the second sample period for all plots combined (Figure 2) and for each vegetation type separately (Figure 3). If this temporal change were caused by the Horseshoe Two Fire, we would expect shifts only for burned plots—a prediction supported by the results, with mean species richness decreasing significantly at low and high severity and marginally insignificantly at moderate severity (Figure 4). Richness exhibited a hump‐shaped relationship with dNBR, but this was, unexpectedly, true for both the prefire (i.e., before any fire at all) and the postfire data, with no significant interaction with timestep (Table 1, Figure S2). The change in the number of species for each plot over time (prefire minus postfire) was not significantly related to dNBR for all plots combined (*p* > .10 data not shown) or for any of the vegetation types analyzed separately, although a marginally insignificant linear decline was found for piñon woodlands (Figure S3). Species richness was significantly shaped by elevation (humped‐shaped polynomial) and TRI (curvilinear increase) but not by TRMI (Table 1, Figure S2)—a relationship that was true for both prefire and postfire, with no interaction between timestep and topography.

Mean evenness increased from the first to the second sample period for all plots combined (Figure 2). This increase occurred for pine–oak forest and piñon woodland but not for juniper woodland (Figure 3). Evenness increased for all fire severity levels, including unburned plots, although the relationship was marginally insignificant for moderate severity (Figure 4). The relationship of evenness to dNBR changed significantly over the two sample periods, exhibiting a hump‐shaped relationship for prefire and a curvilinear increase for postfire (Table 1, Figure S3). For both sample periods, evenness was significantly shaped in a curvilinear fashion by elevation and TRI but not by TRMI (Table 1, Figure S2).

Mean inverse Simpson index increased from the first to the second sample period for all plots combined (Figure 2). This increase occurred for pine–oak forest and juniper woodland but not for piñon woodland (Figure 3). This index increased significantly only for high‐severity plots, although an increase for moderate severity was marginally insignificant (Figure 4). The inverse Simpson index exhibited a hump‐shaped relationship with dNBR, but this was unexpectedly true both for the prefire (i.e., before any fire at all) and for the postfire data, with no significant interaction with timestep (Table 1, Figure S2). The Simpson index changed in a curvilinear manner with elevation (hump‐shaped) and TRI (increasing) but was not significantly related to TRMI (Table 1, Figure S2).

### 3.4 Beta diversity (β)

For “pairwise plots” (see *Methods*), adonis2 tests revealed that dissimilarity increased significantly from the prefire to the postfire sample for total β, species turnover, and nestedness (Figure 2). Species turnover contributed much more to total β than did nestedness: Mean distance from centroids were 0.387 for turnover and 0.070 for nestedness for prefire data and 0.445 and 0.093, respectively, for postfire data. Total β and turnover both increased for juniper woodlands and piñon woodlands tested separately but not for pine–oak forest (Figure 3). Nestedness increased for juniper woodlands and pine–oak forest but not for piñon woodlands. Total β and turnover exhibited significant temporal and spatial trends, more so than did nestedness. If fire were associated with the temporal increase in β, we would expect β to increase over time for burned but not for unburned plots—a hypothesis supported for total and turnover results, but not for nestedness, which increased for burned and unburned plots alike (Figure 4). For both prefire and postfire data, total β declined in a curvilinear manner with elevation, increased linearly with TRMI, and declined moderately with TRI (Table 2, Figure S4).

**TABLE 2:** Statistical results for beta diversity (total, turnover, and nestedness) in relation to continuous fire severity (dNBR) and the topographic variables elevation, topographic relative moisture index (TRMI), and terrain ruggedness index (TRI). (a) Final models from adonis tests (Vegan R package) with Sorensen dissimilarity calculated pairwise among all plots (*n* = 138) separately for prefire and postfire timesteps. (b) Final models from multiple regression for matched plots (prefire‐postfire dissimilarity for each of 138 plots); since timestep is embedded into these values, analyses do not separate prefire and postfire. Table provides *F* values for (A) and *t*‐values for (b), with statistical significance (*p < .05, **p < .01, and ***p < .001). Polynomial models were tested for topographic variables and retained when significant (*p* < .05)

(a) Paired plots							
Model	Factor	Prefire			Postfire		
		Turnover	Nested	Total	Turnover	Nested	Total
Fire severity	dNBR	8.8***	1.0	6.8***	4.7**	1.0	5.3***
	dNBR^2^	10.7***	12.7***	10.1***	5.4**	NS	5.2***
Topography	Elevation	47.7***	0.7	33.1***	28.3***	0.9	22.0***
	Elevation^2^	6.9**	NS	8.5**	6.7***	NS	8.5***
	TRMI	13.6***	1.0	9.7***	7.6***	0.9	5.0**
	TRI	1.9	1.0	2.5*	4.4**	1.1*	4.3**

(b) Matched plots				
		Turnover	Nested	Total
Fire severity	dNBR	1.4	0.9	2.3*
Topography	Elevation	−2.6**	−2.9**	−3.3**
	Elevation^2^	−2.7**	2.9**	NS
	TRMI	−0.7	−0.2	−0.9
	TRI	1.5	−0.9	2.5*

For “matched plots,” the timestep component is embedded in the individual plot prefire/postfire dissimilarities. Mean dissimilarity differed among vegetation types for total β and turnover, but not for nestedness (Figure 3). For total and turnover β, juniper woodland plots changed more than those in piñon woodland, but other pairwise vegetation‐type comparisons were not significant. If fire were a driver of temporal changes in β, we would expect higher prefire/postfire dissimilarity for burned than unburned plots for β—a hypothesis supported for total β (Figure 4). In fact, matched plot beta diversity increased with dNBR up to high severity, at which point it declined slightly (Figure S4). When plot dissimilarity versus dNBR was analyzed separately for each vegetation type, this relationship was significant only for pine–oak forest, which exhibited the same curvilinear relationship of β with dNBR as found for all plots (Figure S3). Finally, plot dissimilarity decreased from low to high elevation for all β components, increased with TRI for total only, and was unrelated to TRMI (Table 2, Figure S4).

For the “Mantel test,” one analysis supported the hypothesis that pyrodiversity begets β among plant communities, but another analysis did not. As dissimilarity in fire severity between two plots increased so did the amount of change in woody plant community dissimilarity from before to after the Horseshoe Two Fire (Mantel *r* = 0.12, *p* = .001). These same pairwise plot contrasts in fire severity were not, however, correlated with postfire plot dissimilarities in communities alone (Mantel *r* = 0.04, *p* = .12). We rejected the null hypothesis that these matrix relationships were artifacts of correlated effects of physical distances among plots (Mantel *r* = 0.02, *p* = .16). Including distance as a second variable in partial Mantel tests also did not change the outcomes of the original analyses.

### 3.5 Contributions of alpha and beta diversity to gamma diversity

For the prefire sample, γ was 37, α 6.7, and Whittaker's β 5.5. In the postfire sample, γ was 49, α 5.4, and Whittaker's β 9.1. In other words, α contributed more than β to landscape scale γ in the first sample, which reversed for the second sample.

## 4 DISCUSSION

Anthropocene fire regimes are catalyzing major shifts in plant community structure, function, and diversity across the world (Bowman et al., 2020). We detected changes in woody plant species diversity in Chiricahua National Monument from 2002–2003 to 2017–2018, a time span intersected by the Horseshoe Two Fire of 2011 and characterized by increasing aridification throughout the southwestern USA (Ault et al., 2016). The key results were that gamma species richness (γ) increased by nearly 1/3, alpha richness (α) declined by 1/5, and beta diversity (β) increased nearly by twofold. As a result, the contributions of α and β to γ reversed their order from the first sample (higher for α) to the second (higher for β). We do not know whether these results apply also to the nonwoody component of plant communities in Chiricahua National Monument. Anecdotal observations suggest a substantial increase in the abundance and diversity of graminoids and forbs after the Horseshoe Two Fire, but we lack quantitative data to assess this possible response.

Several lines of evidence tie these temporal changes directly to the Horseshoe Two Fire for all three scales of species diversity. γ, α species richness, inverse Simpson, total β, and β turnover all shifted from before to after the fire in burned but not unburned plots. The increase in γ, a common result for ecosystems with fire‐adapted species (He et al., 2019; Pausas & Ribeiro, 2017; Perry et al., 2011; Romme et al., 2016), is not surprising for Chiricahua National Monument, given that wildfires have been largely excluded for more than a century in vegetation that historically experienced frequent fire. In our 138 plots, only three species disappeared, while 13 newcomers appeared—primarily cacti indicative of drier conditions and shrub species that thrive in exposed areas, both of which became more prevalent in the wake of the wildfire. The negative impact of the Horseshoe Two Fire on α species richness differs from most studies of plants, which have usually detected positive effects of fire (e.g., Burkle et al., 2015 [forbs and graminoids]; He et al., 2019; Miller & Safford, 2020; but see Collins et al., 2007; Burkle et al., 2015 [woody plants]). The significant increase in the inverse Simpson index aligns more closely with the bulk of those studies, but that result is difficult to interpret given that this index incorporates not just richness but also evenness, which increased in both burned and unburned plots (i.e., independent of the fire).

The generally positive effect of the Horseshoe Two Fire on β is similar to results from studies across a wide range of taxa (He et al., 2019; Miller & Safford, 2020; Myers et al., 2015), although neutral or negative outcomes are common as well (He et al., 2019; Miller & Safford, 2020; Reilly et al., 2006; Richter et al., 2019). β in our study stemmed chiefly from species turnover rather than nestedness, as did significant spatial and temporal patterns. In other words, along fire and topographic gradients, as well as across the two sample periods, woody plant communities tended to exhibit wholesale replacement of species (turnover) rather than shifts in which smaller local assemblages were subsets of larger ones (nestedness). The role of species turnover across topographic gradients accords well with the famously high level of terrain ruggedness and tightly packed communities of Chiricahua National Monument (Poulos et al., 2007) and throughout the Sky Islands (Barton, 1994; Coblentz & Riitters, 2004; Niering & Lowe, 1984; Villarreal et al., 2019; Whittaker et al., 1968). That the Horseshoe Two Fire depressed α species richness but amplified β suggests that fire did not act uniformly in decreasing local richness, but instead filtered out species differentially among plots, promoting variation in postfire assemblages, nearly entirely through the enhancement of species turnover.

The intermediate disturbance hypothesis (IDH) in the context of fire has focused largely on α species richness (Connell, 1978; Huston, 2014; Sousa, 1979), but has also been proposed for β and γ (He et al., 2019). We found support for the IDH for fire severity but only for γ, which peaked at moderate severity. This pattern appears to have stemmed from both higher species extirpation and lower establishment of novel species associated with higher severity fire, probably a result of the extreme conditions in those plots during and after the Horseshoe Two Fire. Outcomes vary widely regarding γ and the IDH, with Richter et al. (2019) supporting the IDH for understory plant communities in the Sierra Nevada, some studies finding no relationship (Miller & Safford, 2020), and others arguing that the positive effect of fire on landscape‐scale plant diversity generally increases linearly along fire gradients (He et al., 2019; Pausas & Ribeiro, 2017).

In contrast to γ, neither α nor β exhibited unambiguous support for the IDH. β increased for all fire severity classes, although the continuous relationship with dNBR exhibited a slight decline at very high severity. α exhibited a hump‐shaped relationship with fire severity, but the same pattern occurs for the results before the fire (Figure S2). These parallel relationships stem not from any impact of fire, of course, but instead from the sharp decline in fire severity with elevation (*r* = 0.43) and an underlying hump‐shaped relationship of diversity with elevation. Only by examining changes in diversity from pre‐ to postfire in burned versus unburned plots were we able to establish that alpha diversity and fire severity exhibited no clear relationship. This pattern demonstrates the importance of coupling pre‐ and postfire sampling, but also reveals a weakness of our study: the lack of experimental treatments in which factors such as fire severity and elevation can be investigated independently.

Although the IDH has been rarely tested for β (see Burkle et al., 2015; Richter et al., 2019 for mixed results), many studies support the proposition for α (DeSiervo et al., 2015; Heydari et al., 2017; He et al., 2019; Miller & Safford, 2020; Morgan et al., 2015; Richter et al., 2019; Stevens et al., 2015; Strand et al., 2019; but see Schwilk et al., 1997). Three factors might explain the negative impact of fire on α and lack of support for the IDH in our results. First, Huston (1994, 2014; see also Burkle et al., 2015) argued on theoretical grounds that a hump‐shaped diversity–disturbance relationship should occur primarily in more productive sites, where disturbance alleviates interspecific competition and promotes species coexistence, rather than in unproductive areas, such as the dry, hot sites in our study area, where interspecific competition may be less pronounced to begin with. The few tests of this IDH‐productivity hypothesis for fire have produced mixed results (Burkle et al., 2015; Miller & Safford, 2020; Strand et al., 2019). Second, more than a century of fire exclusion in Chiricahua National Monument may have reduced the population sizes of less common, fire‐associated species, resulting in lower and more variable seed rain of these species at the local scale after the fire. Moreover, regeneration of these species after the Horseshoe Two Fire, especially from seed, might have been constrained by the long‐term drought that started in the 1990s and was especially extreme postfire in 2011 (Williams et al., 2014). These three scenarios together could translate into the input of relatively few seeds with low establishment probabilities, leading to deficient replacement of species extirpated from local assemblages by the Horseshoe Two Fire, and a lack of positive response to the reduction in local competition assumed by the IDH.

We found mixed support for the hypothesis that pyrodiversity promotes β, a relationship confirmed in many studies of plants (Burkle et al., 2015; Freeman et al., 2019; He et al., 2019; Heydari et al., 2017; Myers et al., 2015; Perry et al., 2011)—but not all (Masunga et al., 2013; Reilly et al., 2006). While our results do not explain whether variation in fire severity led to higher β because of differential fire‐induced mortality or postfire regeneration, the number of novel species after the fire far outstripped the number of extirpated species, suggesting a role for newfound species regeneration in the wake of fire. The decline in α species richness, however, points to the potential importance of differential species mortality across plots. Plots subject to high fire severity, in particular, were markedly transformed by fire‐induced mortality of nearly all stems, massive resprouting of oaks and shrubs, and minimal regeneration of fire‐resistant conifers that rely on establishment by seed. These plots diverged significantly from those experiencing lower fire severities. In Cave Creek Canyon, on the east side of the Chiricahua Mountains, Barton and Poulos (2018; see also Minor et al., 2017) documented such conversion of structurally complex Madrean pine–oak forests to sprouting shrublands after high‐severity fire—a pattern found increasingly across the Southwest (Coop et al., 2020; Falk, 2013; Guiterman et al., 2018). The same process appears in part to have significantly shifted species diversity patterns in Chiricahua National Monument after the Horseshoe Two Fire.

A recent review by Miller and Safford (2020) demonstrated that both α species richness and β are generally highest at fire severities typical of the historical fire regimes of plant communities rather than strictly following the IDH. Ecosystems characterized historically by frequent, surface fire tended to exhibit the highest plant diversity at low‐to‐moderate fire severity, whereas diversity for areas typically experiencing infrequent, stand‐replacing fires often peaked at higher fire severity. Ecological filtering for compatibility of species to fire regime is the assumed driving force underlying these patterns. In our study, pine–oak forest was historically characterized by frequent, low‐severity surface fires (Barton et al., 2001; Swetnam & Baisan, 1996; Swetnam et al., 1989), whereas juniper and piñon woodlands experienced a mixed‐severity fire regime that included high‐severity, stand‐replacing fires (Baisan & Morino, 2000). If historical fire regime shaped the responses of species diversity to fire, we would thus expect divergent responses to fire between pine–oak forest and the other two vegetation types. The results do not support this prediction: The three vegetation types exhibited similar decreases in α species richness and increases in total β over time when burned plots were analyzed separately (*p* < .01; data not shown). Moreover, species richness and pre‐/postfire dissimilarities showed significant continuous relationship with dNBR only for pine–oak forest, for which β peaked at nearly the highest fire severity, contrary to what would have been predicted given its historical low‐severity fire regime.

At least two factors might have mitigated the role of historical fire regime in response to these vegetation types. First, although Miller and Safford (2020) cite evidence for the homogenizing effect of high‐severity fire, a century without fire may well have had the same impact on the vegetation of Chiricahua National Monument, especially pine–oak forest, which was transformed over a century of fire exclusion from an open woodland to a much denser, even light‐limited, forest (Taylor et al., 2021). It is not surprising that fire of any severity may have injected heterogeneity onto this vegetative canvas, promoting β. Second, the species pool governing pine–oak forest includes typical fire‐resistant species that regenerate only from seed (e.g., *Pseudotsuga menziesii* and *P. arizonica*) and thus, as assumed by Miller and Safford (2020), respond poorly to high‐severity fires (Barton, 2002; Barton & Poulos, 2018). Other species common in that vegetation type, however, exhibit multiple traits, such as thick bark, resprouting capacity, and serotinous cones (e.g., *P. leiophylla*), that are adaptive in the context of multiple fire severities. Poulos et al. (2018) argued that such multiple strategies might indicate a complex evolutionary history with respect to the fire regimes experienced by these species. An important assumption of the historical fire regime hypothesis that bears further inspection, therefore, is the extent to which species traits are the legacy of recent historical fire regime versus a longer, more complex evolutionary heritage.

The evenness component of α and the nestedness component of β exhibited temporal changes that were independent of fire; that is, these metrics changed in both burned and unburned plots over the 15‐year period. While this study documents the effects of wildfire on woody plant diversity in Chiricahua National Monument, long‐term drought stress may also be influencing contemporary woody plant diversity dynamics at this site. The region has experienced severe moisture deficits since the 1990s (Ault et al., 2016; Barton & Poulos, 2018; Cook et al., 2014, 2015). Restricted soil moisture and pronounced vapor pressure deficit have caused a wide range of recent ecological changes in the region, including larger, more intense wildfires (Abatzoglou & Kolden, 2013; Abatzoglou & Williams, 2016; Singleton et al., 2019; Williams et al., 2019), pronounced tree mortality (Allen et al., 2010; Williams et al., 2013), and shifts in community composition (Coop et al., 2020; Falk, 2013). We cannot rule out the possibility that moisture stress amplified fire‐associated impacts even in burned plots (Barton & Poulos, 2018; Poulos et al., 2020). The diversity effects of fire comprise the sum of mortality imposed by burning and postfire plant regeneration from seed and resprouting. Intensified moisture stress acting especially on postfire seed germination and establishment may well have acted synergistically with fire in shaping the diversity patterns described in this paper, an argument made also for changes in species composition after the Horseshoe Two Fire (Barton & Poulos, 2018; Poulos et al., 2020). The possible mechanisms connecting fire, drought, and diversity are unclear at this point and deserve further investigation.

Independent of time and fire, topography strongly influenced species diversity in Chiricahua National Monument. In fact, the relationships of diversity indices with topographic variables changed little from before to after the fire, suggesting that topography is an intrinsic regulator of diversity at this site, regardless of the effects of fire. Elevation is a complex master environmental variable controlling the structure, composition, and processes of Sky Island ecosystems (Barton, 1994; Marshall, 1957; Niering & Lowe, 1984; Poulos et al., 2007; Poulos & Camp, 2010; Sawyer & Kinraide, 1980; Shreve, 1915; Whittaker & Niering, 1975). From lower to higher elevation, temperature decreases and moisture increases; other key environmental variables change as well along this gradient (Barton, 1994; Shreve, 1915; Vivoni et al., 2007; Whittaker et al., 1968). α peaked at intermediate elevations and exhibited sharp reductions toward both lower and higher elevations in our study, which is similar to past studies of Chiricahua National Monument (Poulos et al., 2007) and other Sky Island ranges (Whittaker & Niering, 1975). In contrast, β decreased with elevation, suggesting higher levels of habitat heterogeneity at lower elevations. All three α metrics increased with increasing terrain ruggedness (TRI), which measures the degree of topographic complexity at the plot scale. Higher levels of α in more rugged plots likely arise from increased microhabitat heterogeneity and favorable conditions for a wider array of species than in more homogeneous terrain. Coblentz and Riitters (2004) found a similar relationship at the regional scale, attributing the pronounced biodiversity of the Sky Island ranges of the Southwest USA and northern Mexico to the physical complexity of the mountains (see also Felger & Wilson, 1995).

## 5 CONCLUSION

Over the past several decades, temperatures have risen (Gonzalez et al., 2018), moisture availability has declined (Cook et al., 2015), and forest fire activity has increased throughout western North America (Coop et al., 2020; Dillon et al., 2011; Singleton et al., 2019; Westerling, 2016). These changes have depressed postfire tree regeneration (Stevens‐Rumann & Morgan, 2019; Mantgem et al., 2018), compromised forest resilience (Coop et al., 2020; Hessburg et al., 2019), and converted previously conifer‐dominated stands to nonconifer vegetation (Barton & Poulos, 2018; Coop et al., 2020; Guiterman et al., 2018; Minor et al., 2017). The impact of the Horseshoe Two Fire on species diversity in Chiricahua National Park was relatively benign, comparatively. A key impact of the fire, in fact, appeared to be increased landscape heterogeneity with respect to fire history, leading to higher levels of beta diversity. This result should perhaps not be surprising for piñon woodland and juniper woodland, which historically experienced mixed‐severity fire regimes in CHIR, including large areas of high‐severity fire (Baisan & Morino, 2000). Although the higher temperatures and drier conditions of the 21st century depart from historical patterns for the Chiricahua Mountains, the fire pattern imposed by the Horseshoe Two Fire may not differ substantially from infrequent landscape‐scale fire events in the past for these vegetation types (Baisan & Morino, 2000). The results are less explicable for Madrean pine–oak, for which the severity of the fire departed strongly from historical fire regime norms.

From a broader conservation and management perspective, the quantitative metrics suggesting a benign impact of the Horseshoe Two Fire on biological diversity may be misleading. Although the number of species across all plots increased, two ecologically important species—*Pinus arizonica and Pseudotsuga menziesii*—disappeared from the plots (but not the entire park), a result of fire‐induced mortality and lack of regeneration from seed after the fire (Barton & Poulos, 2018; Taylor et al., 2021). Conifer species such as these are foundational elements of the functional and structural complexity of these forests. Despite their replacement by even more shrub species, the loss of these two conifers undoubtedly cascades to other trophic levels and ecosystem processes. The population of the endemic subspecies of the Mexican fox squirrel (*Sciurus nayaritensis chiricahuae*), for example, nests preferentially in sites with large conifers that have experienced low‐severity fire (Doumas & Koprowski, 2013). Although research supports the hypothesis that this vegetation‐type conversion will likely persist (Barton & Poulos, 2018; Guiterman et al., 2018; O’Connor et al., 2020), future episodic regeneration of these conifers under favorable conditions remains a possibility.

Projections call for continuing and even heightened drought (Ault et al., 2016; Wilder et al., 2013) and further intensification of fire in the Southwest (Abatzoglou & Williams, 2016; Kitzberger et al., 2017), putting the woodlands and forests of the Sky Islands at increased risk (Coop et al., 2020; O’Connor et al., 2020; Parks et al., 2019; Yanahan & Moore, 2019). Although the impact of the Horseshoe Two Fire on species diversity was modest in this context, the pattern documented in our study might not replay in future fires for at least two reasons. First, the projected meteorological conditions may increase both the prevalence and frequency of higher severity fire to the extent that species regenerating from seed will not have sufficient time to complete their life cycles between fire events (the so‐called “interval squeeze” problem; Enright et al., 2015). Second, hotter, drier, more fiery conditions are likely to further narrow the window of conditions favorable to postfire regeneration of some species, even if seeds are available (Coop et al., 2020). These two impacts could consequently shift conditions outside the range to which much of the species pool is adapted (Johnstone et al., 2016; Guiterman et al., 2018; Coop et al., 2020; O’Connor et al., 2020; Miller & Safford, 2020), amplifying impacts on ecosystems, including the conversion of complex conifer–hardwood forests to simpler, more resilient nonconifer vegetation (Coop et al., 2020; Falk, 2013). Such environmental changes might, moreover, reduce species diversity at both local and landscape scales, as species pools become constrained by repeated fires and less favorable regeneration conditions (Falk, 2013; O’Connor et al., 2020; Yanahan & Moore, 2019).

Restoring the woodlands and forests described in this paper to pre‐Anthropocene historical norms may no longer be realistic, given the degree of current and projected ecological transformation (Coop et al., 2020; Falk et al., 2019; McWethy et al., 2019). As argued by Keeley et al. (2019), these novel plant communities may simply be better adapted to the current (and future) hotter, drier environment with more fire activity and will need to be accommodated by land managers. Alternatively, efforts could be made to influence biotic change with the goal of conserving important elements of current ecosystems that engender species and functional diversity across all taxa. Interventions such as strategic forest thinning, prescribed fire, protection of refugia for sensitive species, and restoration planting have shown promise in achieving these goals (Laushman et al., 2020; Strom & Fulé, 2007; Villarreal et al., 2020), but could be enhanced by more comprehensive scientific and social–ecological underpinnings (Coop et al., 2020; Falk et al., 2019; Gregg & Marshall, 2020; McWethy et al., 2019).

## CONFLICT OF INTEREST

The authors report no conflict of interest in connection with this study and article.

## AUTHOR CONTRIBUTION

**Andrew M. Barton:** Conceptualization (equal); Data curation (equal); Formal analysis (lead); Funding acquisition (equal); Investigation (equal); Methodology (equal); Project administration (equal); Resources (equal); Software (equal); Supervision (equal); Writing‐original draft (lead). **Helen Poulos:** Conceptualization (equal); Data curation (equal); Formal analysis (supporting); Funding acquisition (equal); Investigation (equal); Methodology (equal); Project administration (equal); Resources (equal); Software (equal); Supervision (equal); Writing‐review & editing (supporting).

## Supporting information

Fig S1

Fig S2

Fig S3

Fig S4

## Data Availability

The data that support the findings of this study are openly available in Dryad at https://doi.org/10.5061/dryad.5x69p8d3w
