# Supplementary figures and images for: Wildfire and topography drive woody plant diversity in a Sky Island mountain range in the Southwest USA
Source: Ecol Evol. 2021 Oct 5;11(21):14715–32. doi: 10.1002/ece3.8158 (PMC8571633; doi:10.1002/ece3.8158)

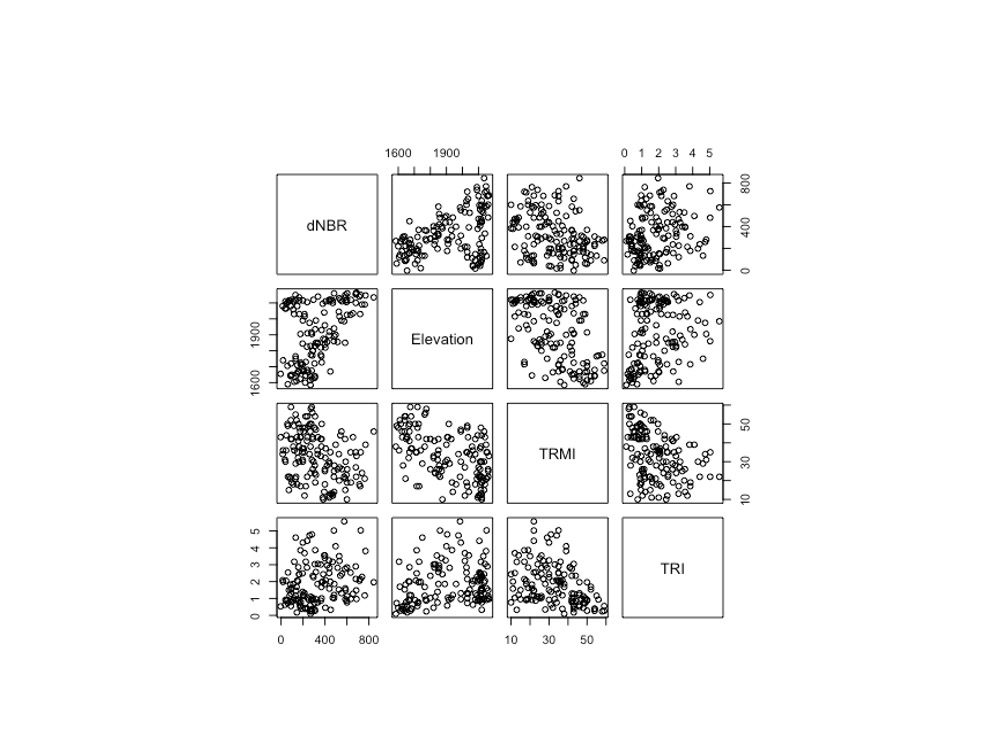

Supplement: Supplementary file 1 — Fig S1 [file ECE3-11-14715-s002.jpg]

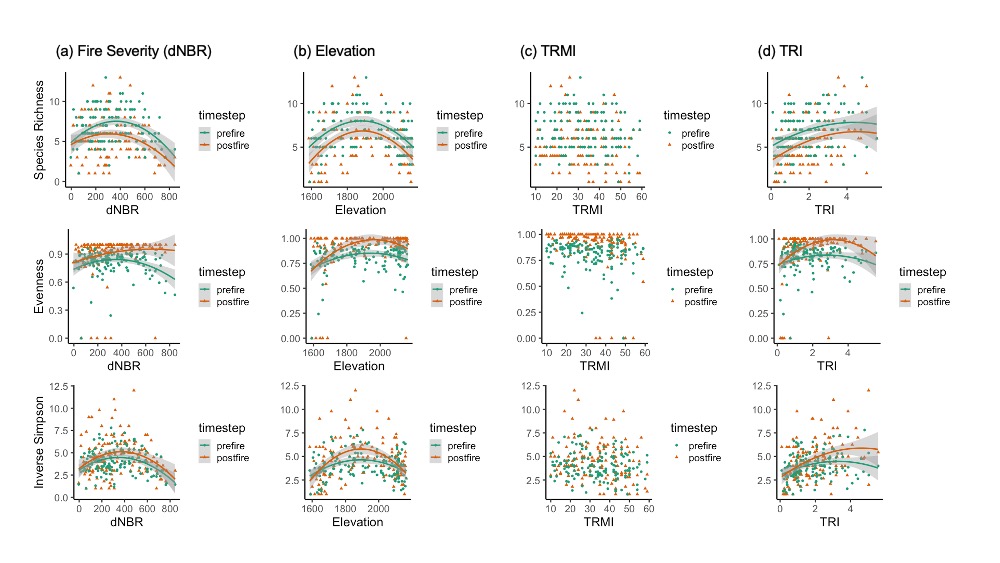

Supplement: Supplementary file 2 — Fig S2 [file ECE3-11-14715-s001.jpg]

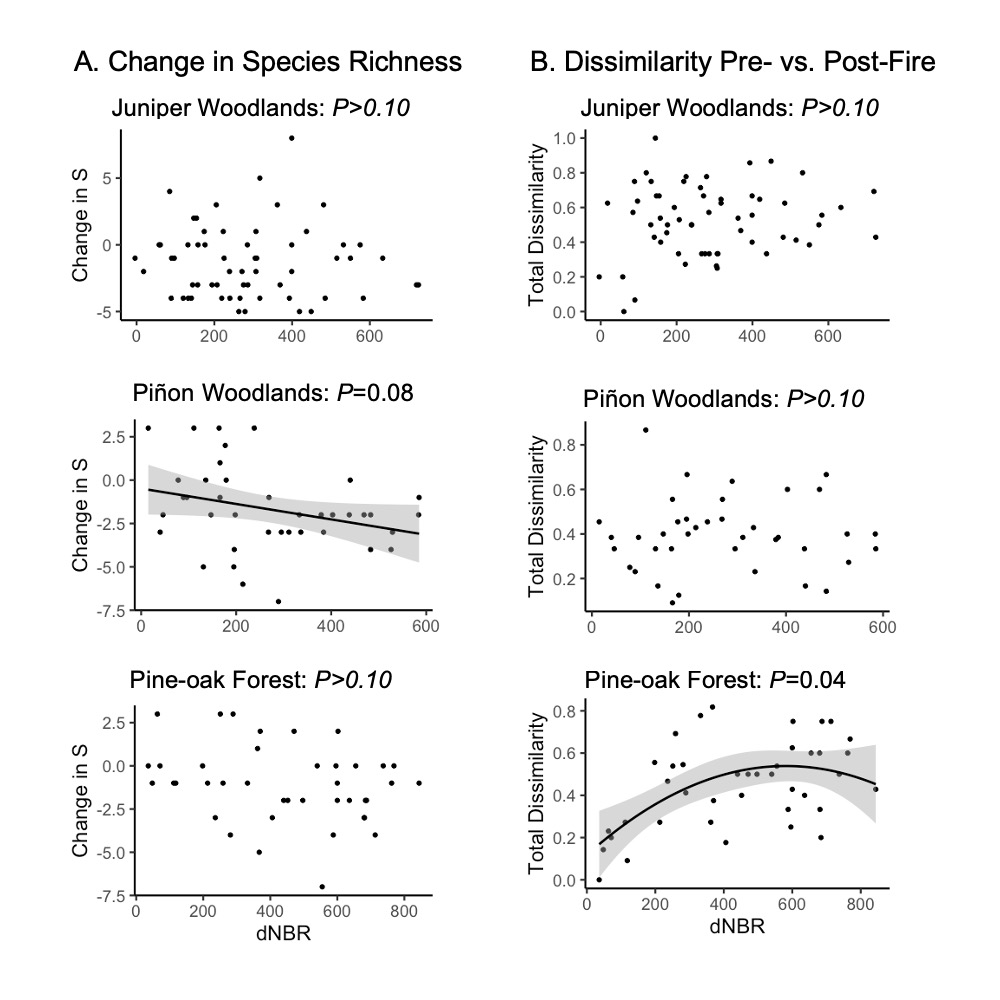

Supplement: Supplementary file 3 — Fig S3 [file ECE3-11-14715-s003.jpg]

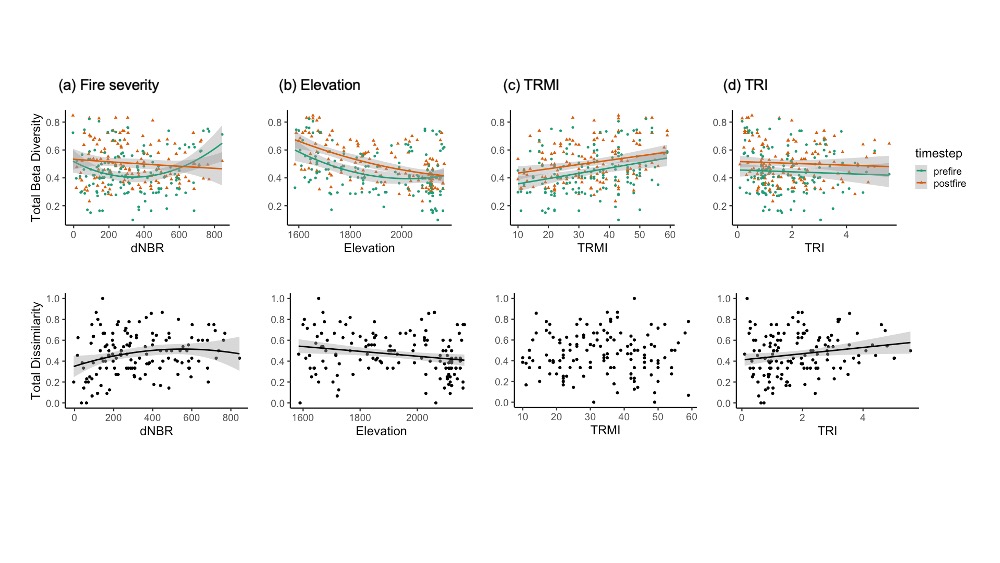

Supplement: Supplementary file 4 — Fig S4 [file ECE3-11-14715-s004.jpg]
